# Plasma or Serum? A Pilot Evaluation of Matrix Selection for Integrated Metabolomics and Exposomics of Clinical Samples

**DOI:** 10.3390/toxics14060494

**Published:** 2026-06-06

**Authors:** Xiaowen Ji, Julian Edwards, Miaomiao Wang, Juan C. Irwin, Binya Liu, Amanda M. Gutierrez, Lin Li, Jeannette C. Lager, Camran R. Nezhat, David K. Stevenson, Tomiko T. Oskotsky, Marina Sirota, Dimitri Abrahamsson, Linda C. Giudice, Tracey J. Woodruff, Joshua F. Robinson, June-Soo Park

**Affiliations:** 1 Environmental Chemistry Laboratory, Department of Toxic Substances Control, California Environmental Protection Agency, Berkeley, CA 94710, USA; julian.edwards@dtsc.ca.gov (J.E.); miaomiao.wang@dtsc.ca.gov (M.W.); june-soo.park@dtsc.ca.gov (J.-S.P.); 2 Department of Obstetrics, Gynecology and Reproductive Sciences, University of California San Francisco (UCSF), San Francisco, CA 94143, USA; juan.irwin@ucsf.edu (J.C.I.); binya.liu@ucsf.edu (B.L.); amanda.gutierrez@ucsf.edu (A.M.G.); lin.li2@ucsf.edu (L.L.); jeannette.lager@ucsf.edu (J.C.L.); dimitri.abrahamsson@ucsf.edu (D.A.); linda.giudice@ucsf.edu (L.C.G.); joshua.robinson@ucsf.edu (J.F.R.); 3 Camran Nezhat Institute, Woodside, CA 94061, USA; camran.nezhat@ucsf.edu; 4 Department of Pediatrics, Stanford University School of Medicine, Stanford, CA 94305, USA; dks750@stanford.edu; 5 Bakar Computational Health Sciences Institute, University of California San Francisco (UCSF), San Francisco, CA 94158, USA; tomiko.oskotsky@ucsf.edu (T.T.O.); marina.sirota@ucsf.edu (M.S.); 6 Department of Epidemiology & Population Health, School of Medicine, Stanford University, Stanford, CA 94305, USA; traceyw@stanford.edu

**Keywords:** serum, plasma, non-targeted analysis, metabolomics, exposomics, matrix selection

## Abstract

Serum and plasma are the most widely used matrices in metabolomics and human biomonitoring studies; however, the optimal matrix for integrated non-targeted analysis (NTA) workflows combining metabolomics and exposomics has not been systematically evaluated. This pilot study applied parallel NTA workflows to paired serum and plasma samples from five individuals to characterize matrix-dependent differences and provide an empirical basis for matrix selection in integrated studies. Three analytical methods were employed: one metabolomic method (Method 1) using Hydrophilic Interaction Liquid Chromatography (HILIC) and Reversed-Phase Liquid Chromatography (RPLC) columns and one exposomics (Method 2) method using an RPLC column, each analyzed in both electrospray ionization (ESI) positive and negative modes. Overall, serum and plasma showed broad similarity, with substantial overlap in detected features and strong linear correlations between paired samples (R^2^ = 0.70–0.87). However, PCA revealed systematic differences between the two matrices along PC1 and PC2, likely attributable to matrix effects arising from coagulation-related compositional changes in serum. For metabolomics, glycerophospholipids, sphingolipids, and acylcarnitines were consistently enriched in serum, attributable to platelet activation and phospholipase release during blood coagulation, consistent with prior reports. In contrast, oxidized fatty acid species were predominantly elevated in plasma, warranting caution in oxylipin-focused studies using serum. For exogenous chemical profiling, the two matrices performed comparably, with 32 out of 36 annotated features showing no significant matrix-dependent differences (*p* > 0.05), including PFAS, pharmaceuticals, and diverse xenobiotics. These findings support the interchangeability of serum and plasma for broad exposomics studies. Overall, while both matrices provided broadly comparable global coverage, plasma may represent a more appropriate matrix for integrated NTA workflows, as it better preserves *in vivo* metabolite composition and minimizes coagulation-induced confounding, though validation in larger cohorts is needed.

## 1. Introduction

Human blood is among the most informative and widely collected biological matrices in clinical and environmental health research. Blood-derived specimens, particularly serum and plasma, offer a comprehensive window into the internal biochemical state of an individual, integrating signals from diet, disease, genetic variation, and environmental exposure [1,2]. As a consequence, serum and plasma have become the dominant matrices in two rapidly expanding fields: clinical metabolomics, which interrogates the endogenous small-molecule landscape to understand disease mechanisms and therapeutic responses [2], and human biomonitoring for exogenous contaminants, which aims to characterize the internal chemical exposome, i.e., the totality of circulating toxic chemicals, pesticides, and environmental contaminants originating from dietary, occupational, and environmental sources that individuals accumulate over their lifetimes [3]. The convergence of these two analytical goals in a single study design is increasingly common, as researchers seek to simultaneously capture both the organism’s metabolic state and its chemical exposure burden from a single blood draw. This integrated multi-analyte approach is analytically efficient and reduces participant burden. However, when both metabolomics and contaminant analysis must be performed on the same sample, the optimal matrix choice remains unresolved.

Serum and plasma are prepared from the same blood draw but fundamentally differ in their collection process. Plasma is obtained by adding an anticoagulant (typically anticoagulants include EDTA and sodium citrate) prior to centrifugation, preserving the native liquid phase, whereas serum requires blood to clot for 15–30 min, during which activated platelets degranulate and release lipid mediators, including TXB2 and 12-HETE, alongside proteins and signaling lipids into the collection tube [4,5]. Previous investigations showed that differences in amino acid and lipid concentrations between the two matrices are systematic and reproducible, with plasma generally demonstrating superior reproducibility [6,7,8,9]. A systematic evaluation of 135 endogenous metabolites across different collection tubes and anticoagulants further demonstrated that exogenous factors, including separation gel ingredients and anticoagulant type, significantly affect metabolite detection, with heparin plasma identified as the optimal matrix for metabolomics research [10]. Consistently, Kaluarachchi et al. demonstrated using untargeted NMR and UPLC-MS that after correcting for inter-individual variation, lysophospholipids and several lipid species were enriched in serum relative to plasma, and that platelet content in plasma subtypes further influenced amino acid and lipid profiles, highlighting the importance of both matrix type and platelet activity on metabolic phenotyping outcomes [11]. This *ex vivo* platelet metabolism confounds the serum metabolome, as demonstrated by Hagn et al. [12], who showed that apparent aspirin-induced downregulation of TXB2 and 12-HETE in serum was an in vitro artifact rather than a true pharmacological effect, while plasma faithfully captured genuine drug responses. On this basis, plasma is recommended as the preferred matrix for clinical metabolomics.

While the serum-versus-plasma debate has been extensively examined in the context of endogenous metabolomics, the analogous question for exogenous contaminant analysis remains strikingly underexplored. Human biomonitoring of environmental pollutants, including persistent organic pollutants (POPs) such as polychlorinated biphenyls (PCBs) and organochlorine pesticides (OCPs), per- and polyfluoroalkyl substances (PFASs), mycotoxins, and other trace-level xenobiotics, has historically been performed in both serum and plasma without systematic justification of matrix choice [13,14]. Serum has traditionally been favored in large regulatory biomonitoring programs (e.g., NHANES) due to historical precedence and the absence of anticoagulant interference [15], while plasma has been increasingly adopted in exposomics research due to its compatibility with biobanked samples, higher throughput collection, and better preservation of labile compounds [16,17]. Critically, no study to date has explicitly investigated whether the platelet activation that occurs during serum preparation also systematically alters the measured concentrations or detection profiles of exogenous small-molecule contaminants. Environmental contaminants are known to interact with plasma proteins and lipids [14,18], and platelet activation releases a broad array of lipids and proteins that alter matrix composition in ways that could plausibly affect contaminant partitioning, apparent binding, and measured concentration. Furthermore, while recent studies have rigorously compared sample preparation methods (e.g., protein precipitation, phospholipid removal, solid-phase extraction) across plasma and serum in the context of chemical exposomics [18,19], these investigations focused on analytical extraction efficiency rather than on the biological differences between matrices arising from coagulation itself, a fundamentally different, and so far, unaddressed, source of potential bias. Beyond analytical considerations, there is an equally important practical motivation: serum and plasma have been collected interchangeably across epidemiological cohorts and biomonitoring programs, often for historical reasons, and it remains unknown whether findings from these studies are directly comparable.

To our knowledge, no study has systematically evaluated whether serum and plasma yield comparable results when metabolomics and exogenous chemical profiling are conducted simultaneously. The most immediate motivation is practical: given that different studies and biobanks have historically collected either serum or plasma, knowing whether the two matrices are interchangeable is essential for cross-study data harmonization. Whether biological differences arising from coagulation further influence measurements, particularly in non-targeted analysis (NTA) workflows where comprehensive, analyte-specific clean-up is absent, adds additional scientific rationale for this comparison. This distinction becomes especially critical in semi-quantitative NTA studies, where matrix-driven differences in chemical background, protein binding, and lipid composition may directly affect detection and apparent abundance of trace-level exogenous compounds. To address this gap, the present pilot study applies an NTA workflow to paired serum and plasma samples from the same individuals, aiming to identify matrix-dependent differences and provide an empirical basis for matrix selection in integrated metabolomics and exposomics studies.

## 2. Materials and Methods

### 2.1. Collection of Serum and Plasma

Blood samples for this study were obtained through the UCSF Human Endometrial Tissue and DNA Bank, after written informed consent, under an approved UCSF IRB protocol. Inclusion criteria are subjects 18–50 years old undergoing treatment for benign gynecologic conditions. Exclusion criteria are known or suspected pregnancy, endometrial hyperplasia and cancer, and significant systemic disease. Five paired serum and plasma samples were collected from female participants at the University of California, San Francisco (UCSF). Participant ages ranged from 37 to 50 years (mean ± SD: 43.2 ± 4.7 years). Blood samples were collected at the time of surgery, including two participants with endometriosis and three undergoing surgeries for other gynecologic indications, providing a consistent perioperative context. For each participant, whole blood was collected simultaneously into an EDTA-treated anticoagulant tube for plasma and an untreated tube for serum preparation. Participants included individuals both receiving and not receiving hormonal therapies at the time of sample collection. Reported hormonal exposures included combined hormonal contraceptives, intrauterine progestin delivery systems, and ovarian suppression regimens. Additional non-hormonal medications (e.g., supplements, analgesics, and other routine medications) were reported but were not evaluated as primary variables in the analysis. Given the paired study design (serum and plasma collected concurrently from the same individual), inter-individual variability related to hormonal status, medication exposure, and baseline physiology was controlled through within-subject comparisons; therefore, therapies were not used as stratification variables.

### 2.2. Sample Preparation

Serum and plasma samples were stored at −80 °C and thawed at room temperature (~20 °C) immediately prior to extraction. Two previously established NTA extraction protocols (including the instrumental method) were applied in parallel: Method 1 for metabolomics [20,21] and Method 2 for exposomics [22], each with some minor modifications. All extracts were analyzed by LC-MS immediately after preparation. Methanol, water, acetonitrile, and isopropanol are LC-MS grade, and ammonium hydroxide, ammonium acetate, formic acid, and acetic acid are HPLC grade (J.T.Baker, Avantor, Inc., Radnor, PA, USA).

#### 2.2.1. Method 1 (Metabolomics)

Each serum or plasma sample (100 µL) was spiked with 100 µL of L-Isoleucine–^13^C_6_ (10 ng/mL in ACN:H_2_O, 1:1, *v*/*v*; Cambridge Isotope Laboratories, Tewksbury, MA, USA) as an internal standard. Protein precipitation and metabolite extraction were performed by adding 1 mL of MeOH:ACN:H_2_O (2:2:1, *v*/*v*/*v*) followed by vortexing for 30 s. Samples were then snap-frozen on dry ice for 1 min, thawed at room temperature, and sonicated for 10 min in a 4 °C water bath. This freeze–thaw–sonication cycle was repeated three times. Samples were incubated at −20 °C for 1 h to facilitate protein precipitation, and then centrifuged at 13,000 rpm for 15 min at 4 °C (Sorvall X4 Pro-MD, Thermo Fisher Scientific, Waltham, MA, USA). The supernatant was transferred and evaporated to dryness at 4 °C under a gentle nitrogen stream (TurboVap, Biotage, San Jose, CA, USA). Dried residues were reconstituted in 100 µL of ACN:H_2_O (1:1, *v*/*v*), sonicated for 10 min, and centrifuged again at 13,000 rpm for 15 min at 4 °C prior to analysis.

#### 2.2.2. Method 2 (Exposomics)

Each serum or plasma sample (100 µL) was spiked with 25 µL of a 500 ng/mL isotope-labeled internal standard mixture containing M2PFOA, M3PFBA, and M6PFDA (Wellington Laboratories, Guelph, ON, Canada), as well as d15-triphenyl phosphate and DL-cotinine-d3 (Cambridge Isotope Laboratories, Tewksbury, MA, USA). Extraction was performed by adding 2 mL of methanol containing 0.1% formic acid (pre-cooled at −18 °C), followed by shaking at 1200 rpm for 15 min (Analog Multi-Tube Vortexer, Fisher Scientific, Hampton, NH, USA) and centrifugation at 3000 rpm for 20 min at 4 °C. The supernatant was collected and concentrated under a gentle nitrogen stream to less than 0.5 mL, transferred to 1.5 mL microcentrifuge tubes, and evaporated to near dryness. Residues were reconstituted in 100 µL of MeOH:H_2_O (1:1, *v*/*v*) and centrifuged for 20 min, and the final supernatant was transferred to autosampler vials for LC-MS analysis.

### 2.3. Instrumental Analysis

Sample analysis was performed using an Agilent 1290 Infinity UHPLC system coupled to an Agilent 6550 iFunnel Quadrupole Time-of-Flight mass spectrometer (Q-TOF-MS; Agilent Technologies, Santa Clara, CA, USA). Each sample was injected twice and analyzed in both electrospray ionization positive (ESI^+^) and negative (ESI^−^) ionization modes.

#### 2.3.1. Method 1 (HILIC and RPLC)

Extracts from Method 1 were analyzed using two complementary chromatographic modes—Hydrophilic Interaction Liquid Chromatography (HILIC) and Reversed-Phase Liquid Chromatography (RPLC). HILIC separation was performed on a Waters ACQUITY UPLC BEH Amide column (1.7 µm, 2.1 × 100 mm, Milford, MA, USA) with mobile phase (A) 100% H_2_O containing 25 mM ammonium acetate and 25 mM ammonium hydroxide and (B) 100% acetonitrile, at a flow rate of 0.5 mL/min. RPLC separation was performed on a Phenomenex Kinetex^®^ C18 column (2.6 µm, 2.1 × 100 mm, Torrance, CA, USA) with mobile phase (A) 100% H_2_O containing 0.01% acetic acid and (B) isopropanol:acetonitrile (1:1, *v*/*v*), at a flow rate of 0.3 mL/min. For both columns, the injection volume was 2 µL and the autosampler was maintained at 4 °C. Twelve metabolite standards were analyzed midway through the sequence for HILIC and RPLC, respectively, to enable recalibration for the MetDNA (Metabolite Identification and Dysregulated Network Analysis) tool [20]. Gradient elution programs are detailed in Supporting Spreadsheet S1.

MS data were acquired on the Q-TOF-MS. Source parameters were set as follows: sheath gas temperature, 400 °C; dry gas temperature, 250 °C; sheath gas flow, 12 L/min; dry gas flow, 16 L/min; and capillary voltage, ±3000 V. Full-scan MS1 spectra were acquired over *m*/*z* 50–1200 at an acquisition rate of 4 spectra/s. Tandem mass spectrometry (MS/MS) data were collected in auto MS/MS mode, with precursor ion selection divided into six sequential mass windows: 60–180, 170–300, 290–450, 440–600, 590–900, and 890–1200 Da. MS and MS/MS acquisition rates were set at 5 and 2 spectra/s, respectively, with a maximum of 2 precursor ions selected per cycle. The MS/MS scan range was set to *m*/*z* 25–1200. Collision energies were set to +20 V and −20 V for positive and negative ionization modes, respectively.

#### 2.3.2. Method 2 (RPLC)

Chromatographic separation was achieved using a reversed-phase EclipsePlus C18 column (2.1 × 100 mm, 1.8 µm particle size; Agilent Technologies, Santa Clara, CA, USA) maintained at 40 °C. A binary solvent system consisting of water (mobile phase A) and methanol (mobile phase B), each containing 5 mM ammonium acetate, was used. The flow rate was maintained at 0.3 mL/min throughout the run. The detailed LC gradient program is provided in Supporting Spreadsheet S1.

The injection volume was 10 µL for all acquisitions. ESI source parameters were as follows: sheath gas temperature, 450 °C; drying gas temperature, 250 °C; sheath gas flow, 11 L/min; drying gas flow, 12 L/min; capillary voltage, ±3000 V; nozzle voltage, 0 V; and nebulizer pressure, 50 psi (ESI^+^) and 45 psi (ESI^−^). For MS1 profiling, all individual samples were analyzed in MS1-only mode over an *m*/*z* range of 50–1200. MS/MS spectra were acquired for each sample using Auto MS/MS mode. The precursor ion *m*/*z* windows were segmented into the same six overlapping ranges as in Method 1. For Auto MS/MS acquisition, the full scan ranges were set to *m*/*z* 50–1200 for MS1 and *m*/*z* 25–1200 for MS/MS, respectively, with a maximum of two precursor ions selected per cycle. Collision energies of 10, 20, and 40 V were applied.

### 2.4. Quality Assurance (QA) and Quality Control (QC)

To minimize time-dependent instrumental variation, serum and plasma samples were arranged in an interleaved injection sequence, alternating between matrices throughout the run, ensuring that any signal drift or carry-over affected both matrices equally and did not confound matrix-dependent differences in the analytical results. Matrix-matched QC samples were prepared separately for each matrix using Human AB Serum (MP Biomedicals, Burlingame, CA, USA) and Human K2EDTA Plasma (Equitech-Bio Inc., Kerrville, TX, USA) as blank matrices. Each blank matrix was spiked with 17 analytical standards (7 in positive mode and 10 in negative mode) at a concentration of 100 ng/mL. These matrix-spiked QC samples were injected at the middle and end of each analytical sequence to monitor instrument sensitivity and stability over time. In addition, unspiked matrix blanks were injected immediately after the instrument blank at the beginning of each batch to assess background interference from the matrix itself. For individual samples, isotope-labeled internal standards (ISTDs) were included in each extraction to monitor extraction consistency within each batch. While ISTDs cannot represent the full chemical diversity detected in NTA workflows, they serve as reliable indicators of extraction reproducibility and ionization stability. For both QC analytes and ISTDs, retention time (RT) shifts were required to remain within 15–30 s and the RSD of peak areas not to exceed 20%. It should be noted that matrix-spiked QC samples were acquired in full-scan MS1 mode only. The signal abundances of spiked analytes in QC samples and ISTDs in individual samples were evaluated using Agilent MassHunter Qualitative Analysis (version 10.0), with a mass accuracy tolerance of 10 parts per million (ppm). The RT, measured mass, and peak area of the ISTDs and QC analytes are reported in Supporting Spreadsheet S2.

### 2.5. Data Processing

#### 2.5.1. Peak Feature Alignment

The raw data files were imported using the MSnbase R package (version 2.23.0) and processed for alignment with the XCMS R package (version 4.4.0). All analyses were performed in RStudio (version 2026.1.1.403) with R (version 4.5.1). The alignment parameters followed the previous method [23]. Briefly, the centWave algorithm was applied with a mass accuracy of 10 ppm, a peak width of 5–30 s, a signal-to-noise threshold of 6, a minimum noise level of 500, and a prefilter requiring at least 3 peaks with a minimum intensity of 3000. Downstream analyses were performed using Python 3.13.11 on the Spyder platform (version 6.1.3) running on Windows 11.

#### 2.5.2. Peak Feature Identification

Chemical identification was performed at two confidence levels following the framework proposed by Schymanski et al. [24]: level 1 (confirmed by authentic standards) and level 2 (matched to spectral databases). However, given the fundamental differences between metabolomics and exogenous chemical profiling, confidence levels were applied differently across the two chemical spaces. For exogenous chemicals, experimental MS/MS spectra were matched against MassBank of North America and MassBank of Europe using the Python package matchms (v0.28.1). Prior to matching, spectra were preprocessed by intensity normalization and *m*/*z* filtering (50–1000). Spectral similarity was assessed using cosine similarity with a threshold of >0.9 and a precursor mass tolerance of 5 ppm, yielding level 2 identifications. Compounds confirmed by authentic standards available in our laboratory inventory were elevated to level 1. For endogenous metabolites, MS/MS spectra and RT matching samples were annotated at level 1 using MetDNA [20], which first annotates initial seed metabolites using a small standard MS^2^ spectral library, matching experimental MS/MS spectra against reference spectra using a dot-product score threshold, with RT confirmed against reference values previously established by this study using authentic chemical standards on the same LC column and instrument conditions. All identification peak features have been listed in Supporting Spreadsheet S3–S8. To minimize misannotation and reduce the risk of phantom metabolites arising from peak alignment artifacts, cases where the same chemical identity was assigned to multiple peak features were manually inspected by extracted ion chromatogram (EIC). Peak shape, retention time consistency, and alignment integrity were evaluated for each candidate, and redundant or artifactual annotations were removed, retaining only the most reliable feature per compound.

#### 2.5.3. Missing Value Imputation

A detection frequency filter was applied independently to each matrix prior to imputation. Peak features detected with non-zero intensity in fewer than 80% of samples within a given matrix were excluded to ensure that only features present in a majority of samples were considered for further analysis. For the remaining features, missing values were imputed using a K-nearest neighbors (KNN) algorithm (k = 5) implemented via the KNNImputer function in the scikit-learn package (1.8.0), which estimates missing values based on the five most similar samples in the feature space. This preprocessing workflow was applied independently for each chromatographic column and ionization polarity combination.

#### 2.5.4. Statistical Analysis

Prior to principal component analysis (PCA), the imputed peak feature matrix was log2-transformed. No additional feature filtering or scaling was applied before PCA computation. PCA was performed on detected peak features by plotting PC1 versus PC2 to visualize matrix-dependent separation between serum and plasma. Linear regression of mean log-transformed abundances between serum and plasma was conducted to assess whether differences between the two matrices were systematic (i.e., a consistent matrix effect) rather than random (i.e., noise or biological variability). For individual identified chemicals, peak feature abundances were log2-transformed and compared between serum and plasma using the Mann–Whitney U test, with a significance threshold of *p* < 0.05. Given the small sample size (*n* = 5 per group), this analysis was exploratory in nature, and multiple testing correction (e.g., Benjamini–Hochberg procedure) was not applied due to concerns about excessive false negatives at this scale. While a two-factor linear regression or fixed-effects model incorporating both matrix and participant as factors could in principle better account for within-subject variability, the limited sample size precluded a robust implementation of such an approach. Results should therefore be interpreted with appropriate caution and viewed as hypothesis-generating rather than confirmatory. Applying false discovery rate correction under these conditions would further reduce sensitivity and risk masking biologically or analytically meaningful matrix differences. The primary aim of this comparison was exploratory, to flag chemicals exhibiting potentially relevant matrix-dependent behavior, rather than confirmatory hypothesis testing.

## 3. Results

### 3.1. All Features

**Figure 1:**
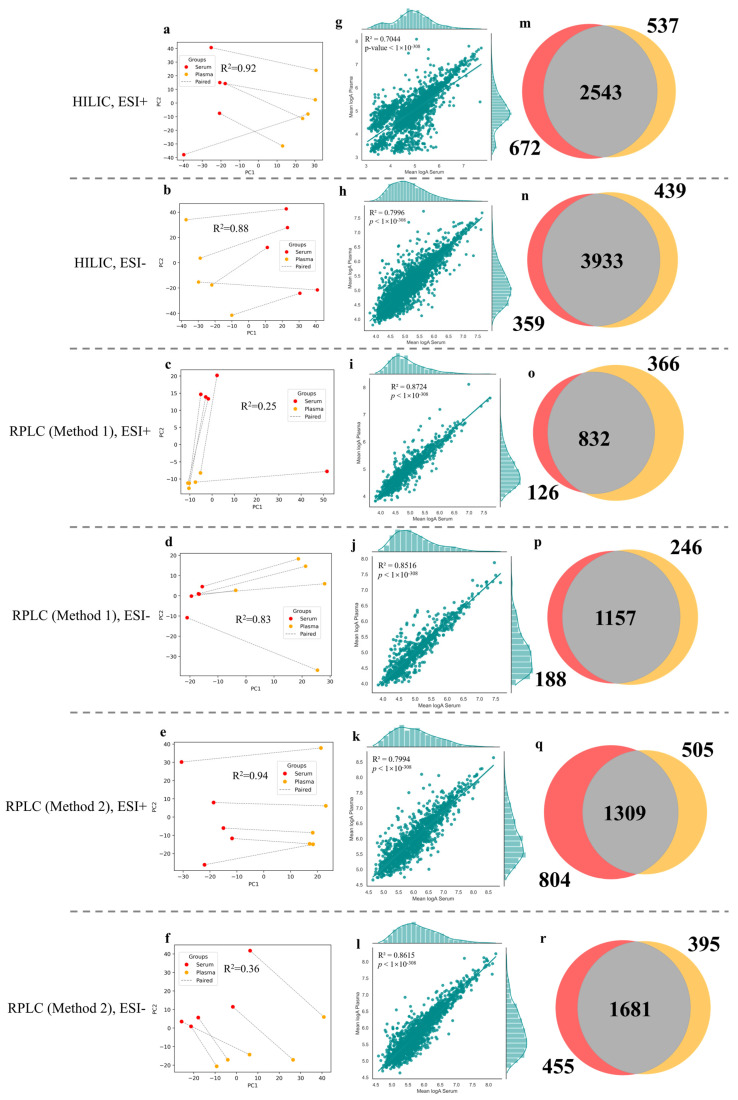
Principal component analysis (PCA) of peak features detected in both serum and plasma with a detection frequency ≥ 80%, colored by sample type (serum vs. plasma), for Method 1 (HILIC column, metabolomics) in ESI^+^ (**a**) and ESI^−^ modes (**b**); Method 1 (RPLC column, metabolomics) in ESI^+^ (**c**) and ESI^−^ modes (**d**); and Method 2 (RPLC column, exogenous chemicals) in ESI^+^ (**e**) and ESI^−^ modes (**f**). The dashed grey lines in the PCA plot indicate paired serum and plasma samples. Linear regression of mean peak feature abundance between serum and plasma across ESI^+^ and ESI^−^ modes for all three methods (**g**–**l**). Venn diagrams illustrating the overlap and unique peak features between serum and plasma for features with a detection frequency ≥ 80% in either matrix (**m**–**r**).

When visualizing the PCA results, PCA plots of PC1 versus PC2 showed separation between serum and plasma samples across most chromatographic methods and ionization modes (Figure 1a–f), with the degree of separation varying across column methods. The first two principal components collectively captured between 37.3% and 57.3% of total variance across all six analytical conditions. Matrix-dependent separation between serum and plasma along PC1 was statistically significant in five of six conditions (Mann–Whitney U test, *p* = 0.0079), with the exception of RPLC ESI^−^ Method 2 (*p* = 0.151). The proportion of PC1 variance attributable to matrix type (R^2^) ranged from 0.25 (RPLC ESI^+^ Method 1) to 0.94 (RPLC ESI^+^ Method 2), with particularly strong matrix-driven separation observed in RPLC ESI^+^ Method 2 (R^2^ = 0.94), HILIC ESI^+^ (R^2^ = 0.92), HILIC ESI^−^ (R^2^ = 0.88), and RPLC ESI^−^ Method 1 (R^2^ = 0.83). The relatively weaker separation in RPLC ESI^−^ Method 2 (R^2^ = 0.36) and RPLC ESI^+^ Method 1 (R^2^ = 0.25) may reflect broader chemical coverage in these conditions, where inter-individual variability contributes more substantially to overall variance. Additionally, we noted a strong linear correlation (R^2^ = 0.70–0.87, *p* < 1 × 10^−308^) for the average log-transformed features between serum and plasma across all methods (Figure 1g–l).

For Method 1 (HILIC column), in the ESI^+^ mode, 2543 peak features were detected in both serum and plasma with ≥80% detection frequency, with 672 features (21% of all features) unique to serum and 537 features (17% of all features) unique to plasma (Figure 1m). In the ESI^−^ mode, 3933 peak features were detected with ≥80% frequency, with 359 features (8% of all features) unique to serum and 439 features (10% of all features) unique to plasma (Figure 1n).

For Method 1 (RPLC column), in the ESI^+^ mode, 832 peak features were detected in both serum and plasma with ≥80% frequency, with 126 features (13% of all features) unique to serum and 366 features (31% of all features) unique to plasma (Figure 1o). In the ESI^−^ mode, 1157 peak features were detected with >60% frequency, with 188 features (14% of all features) unique to serum and 246 features (18% of all features) unique to plasma (Figure 1p).

For Method 2 (RPLC column), in the ESI^+^ mode, 1309 peak features were detected in both serum and plasma with ≥80% frequency, with 804 features (38% of all features) unique to serum and 505 features (28% of all features) unique to plasma (Figure 1q). In the ESI^−^ mode, 1681 peak features were detected in both serum and plasma with ≥80% frequency, with 455 features (21% of all features) unique to serum and 395 features (19% of all features) unique to plasma (Figure 1r). Given the exploratory nature of this pilot study and the limited sample size (*n* = 5), all findings should be interpreted with caution and regarded as preliminary observations warranting confirmation in larger cohorts.

### 3.2. Level 1 Metabolites

**Figure 2:**
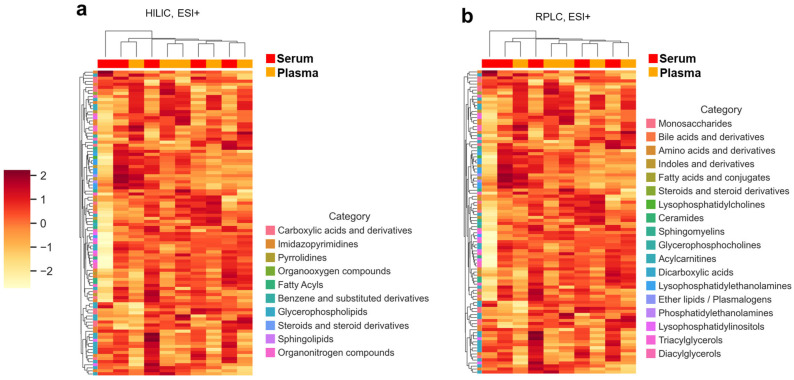
Clustered heatmap of confirmed metabolites (level 1) detected by ESI^+^ in Method 1 using the HILIC (**a**) and RPLC (**b**) columns, comparing serum and plasma samples. Metabolite sub-categories are color-coded separately for each column method.

To characterize the metabolite profiles of serum and plasma, we focused on level 1 confirmed metabolites across both the HILIC (129 metabolites) and RPLC (86 metabolites) columns in Method 1. The clustered heatmap revealed distinct abundance patterns between serum and plasma samples, with samples clustering slightly by matrix type rather than by individual variation for HILIC column (Figure 2a) while no readily apparent clustering was observed for the RPLC column (Figure 2b).

In the HILIC column, a subset of metabolites showed relatively higher abundance in serum compared to plasma. Among the detected sub-categories, glycerophospholipids and sphingolipids showed the most notable abundance differences between serum and plasma, with consistently lower abundance in plasma relative to serum. In contrast, carboxylic acids and derivatives and organonitrogen compounds appeared more uniformly distributed across both matrices.

**Figure 3:**
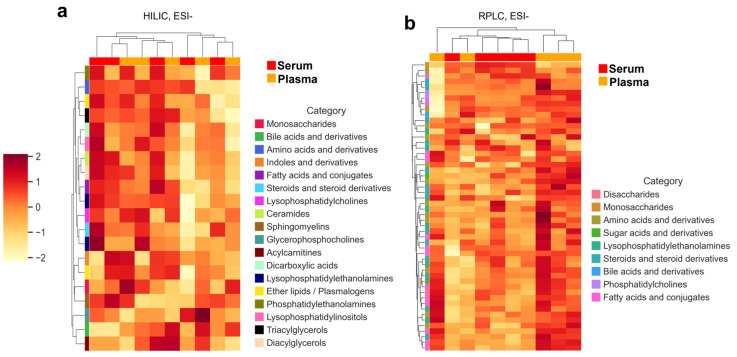
Clustered heatmap of confirmed metabolites (level 1) detected by ESI^−^ in Method 1 using the HILIC (**a**) and RPLC (**b**) columns, comparing serum and plasma samples. Metabolite sub-categories are color-coded separately for each column method.

For ESI^−^ mode, the clustered heatmap of level 1 confirmed metabolites similarly revealed matrix-dependent separation between serum and plasma samples across both column methods. In the HILIC column (Figure 3a), lysophosphatidylcholines (LPCs) showed the most striking abundance difference between serum and plasma, with one distinct cluster of features displaying markedly higher abundance in one matrix relative to the other. Lysophosphatidylethanolamines and sphingomyelins were represented by fewer features but similarly exhibited differential abundance between the two sample types. In the RPLC column (Figure 3b), amino acids and derivatives represented the largest metabolite cluster and showed variable abundance patterns between serum and plasma. Bile acids and derivatives displayed a particularly pronounced abundance difference, with a subset of features showing a near-absent signal in one matrix compared to the other. Fatty acids and conjugates and steroids and steroid derivatives also contributed to the observed separation between serum and plasma.

**Figure 4:**
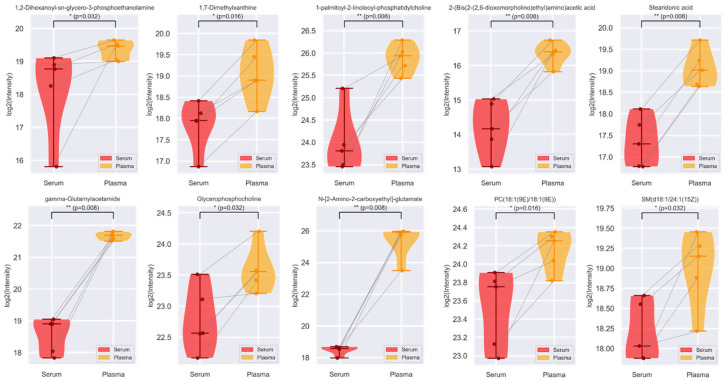
Violin plots of log2-transformed abundance for level 1 confirmed metabolites with significantly higher abundance in plasma compared to serum, as determined by the Mann–Whitney U test (*p* < 0.05) for the HILIC column method. Grey lines connect paired serum and plasma samples. Asterisks indicate statistical significance based on the Mann–Whitney U test (* *p* < 0.05, ** *p* < 0.01).

More specifically, we applied the Mann–Whitney U test to level 1 confirmed metabolites and focused on those with significantly higher abundance in plasma compared to serum (*p* < 0.05) for the HILIC column. A total of 10 metabolites were identified across diverse chemical classes, representing all level-1 confirmed metabolites meeting the significance threshold (Figure 4). Among lipid species, 1-palmitoyl-2-linoleoyl-phosphatidylcholine, PC(18:1(9E)/18:1(9E)), and SM(d18:1/24:1(15Z)) showed relatively higher abundance in plasma, suggesting that certain phosphatidylcholines and sphingomyelins may be preferentially retained in plasma relative to serum. Similarly, 1,2-dihexanoyl-sn-glycero-3-phosphoethanolamine displayed elevated plasma abundance, consistent with possible matrix-dependent differences in glycerophospholipid levels.

Among polar metabolites, gamma-glutamylacetamide, N-(2-amino-2-carboxyethyl)-glutamine, and glycerophosphocholine were markedly higher in plasma. Additionally, 1,7-dimethylxanthine, 2-[bis[2-chloromorpholino]ethyl]amino]acetic acid, and stearidonic acid were also significantly elevated in plasma compared to serum.

**Figure 5:**
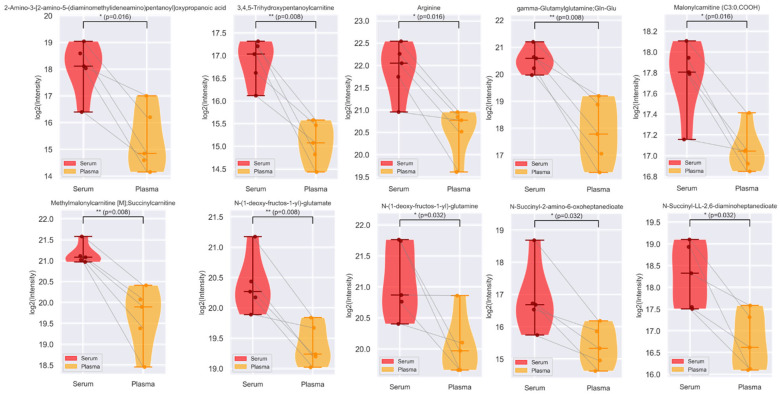
Violin plots of log2-transformed abundance for level 1 confirmed metabolites with significantly higher abundance in serum compared to plasma, as determined by the Mann–Whitney U test (*p* < 0.05) for the HILIC column method. Grey lines connect paired serum and plasma samples. Asterisks indicate statistical significance based on the Mann–Whitney U test (* *p* < 0.05, ** *p* < 0.01).

Conversely, 10 metabolites were found to be significantly higher in serum compared to plasma (*p* < 0.05, Figure 5). These metabolites were predominantly amino acid derivatives and acylcarnitine-related compounds. Specifically, arginine, gamma-glutamylglutamine (Gln-Glu), and 2-amino-3-(2-amino-5-(diaminomethylideneamino)pentanoyl)oxypropanoic acid were markedly elevated in serum, pointing to systematic differences in amino acid metabolism-related metabolites between the two matrices. Similarly, 3,4,5-trihydroxypentanoylcarnitine, malonylcarnitine (C3:0, COOH), and methylmalonylcarnitine/succinylcarnitine showed substantially higher abundance in serum, potentially consistent with platelet-related release during coagulation, though this requires further validation. Among other elevated serum metabolites, N-(1-deoxy-fructos-1-yl)-glutamate, N-(1-deoxy-fructos-1-yl)-glutamine, N-succinyl-2-amino-8-oxoheptanedioate, and N-succinyl-1,7-diaminoheptanedioate were also significantly enriched in serum relative to plasma, suggesting possible matrix-dependent differences in glycated amino acid derivatives and lysine metabolism intermediates.

**Figure 6:**
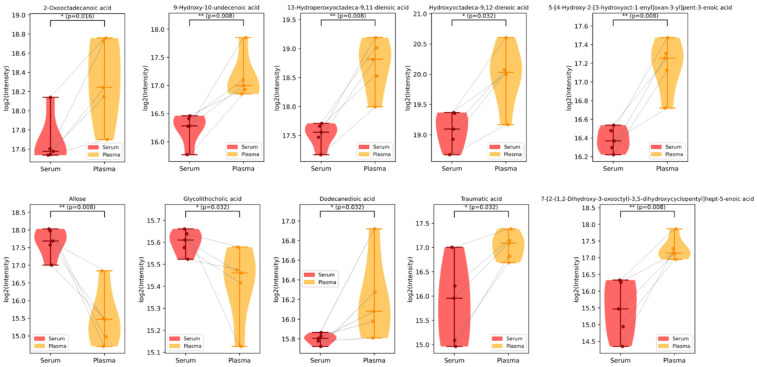
Violin plots of log2-transformed abundance for level 1 confirmed metabolites with significantly different abundance between serum and plasma, as determined by the Mann–Whitney U test (*p* < 0.05) for the method 1 RPLC column. Grey lines connect paired serum and plasma samples. Asterisks indicate statistical significance based on the Mann–Whitney U test (* *p* < 0.05, ** *p* < 0.01).

For the RPLC column, a distinct set of metabolites showed significant abundance differences between serum and plasma (*p* < 0.05, Figure 6). In contrast to the HILIC findings, the differentially abundant metabolites detected by the RPLC column were predominantly fatty acid derivatives and bile acid-related compounds. Specifically, 2-oxooctadecanoic acid, 9-hydroxy-10-undecenoic acid, 1,3-hydroperoxy octadeca-9,11-dienoic acid, hydroxyoctadeca-9,12-dienoic acid, and 3-(4-hydroxy-3-(3-hydroxyoct-1-enyl)oxen-3-yl)pent-3-enoic acid were all significantly higher in plasma compared to serum, suggesting that oxidized and hydroxylated fatty acid species may be enriched in plasma relative to serum. Among metabolites higher in serum, allose, glycolithocholic acid, dodecanedioic acid, traumatic acid, and 7-(2-(1,2-dihydroxy-3-oxooctyl)-3,5-dihydroxycyclopentyl)hept-5-enoic acid were significantly elevated, spanning sugar, bile acid, and oxylipin chemical classes.

### 3.3. Level 1 & 2 Exogenous Chemicals

**Figure 7:**
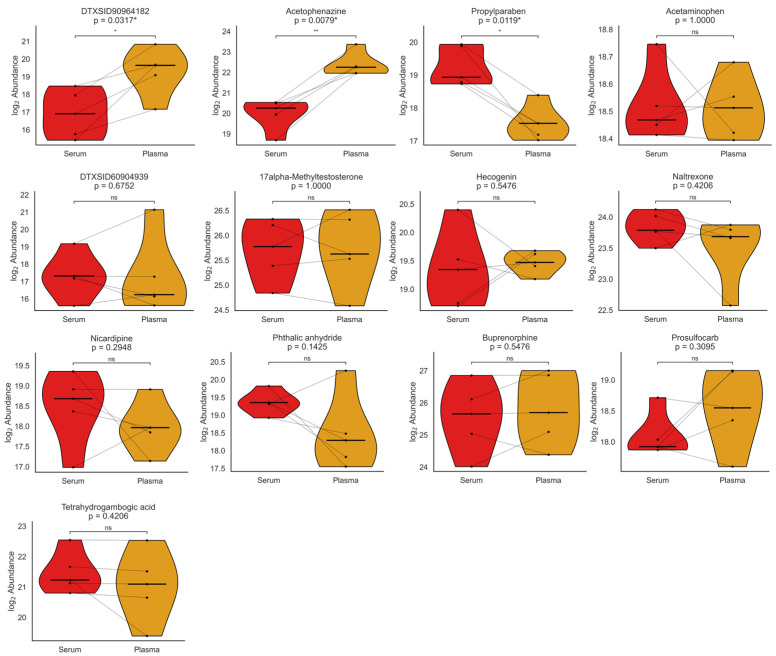
Violin plots of log2-transformed abundance for level 1 and level 2 confirmed exogenous chemicals showing either significant (*p* < 0.05) or no significant difference between serum and plasma, as determined by the Mann–Whitney U test (Method 2, RPLC column, ESI^+^ mode). Acetaminophen was the only level 1 confirmed chemical. Asterisks indicate statistical significance based on the Mann–Whitney U test (* *p* < 0.05, ** *p* < 0.01) and ns indicates not significant.

For exogenous chemicals detected by the RPLC column in Method 2 for ESI^+^ mode, the majority showed no significant difference in abundance between serum and plasma (Figure 7). Among the thirteen exogenous chemicals annotated (levels 1–2), ten, including 17alpha-methyltestosterone, acetaminophen (level 1), buprenorphine, hecogenin, naltrexone, prosulfocarb, DTXSID60904939 (one terpenoid ester), nicardipine, phthalic anhydride, and tetrahydrogambogic acid, showed no significant difference between the two matrices (*p* > 0.05). Acetaminophen, the only level 1 confirmed exogenous chemical, was among those showing no significant matrix-dependent difference. In contrast, two exogenous chemicals (level 2), acetophenazine and DTXSID90964182 (a hydroxylated furochromone), were significantly higher in plasma compared to serum (*p* < 0.05). Only propylparaben was significantly more abundant in serum than in plasma (*p* < 0.05).

**Figure 8:**
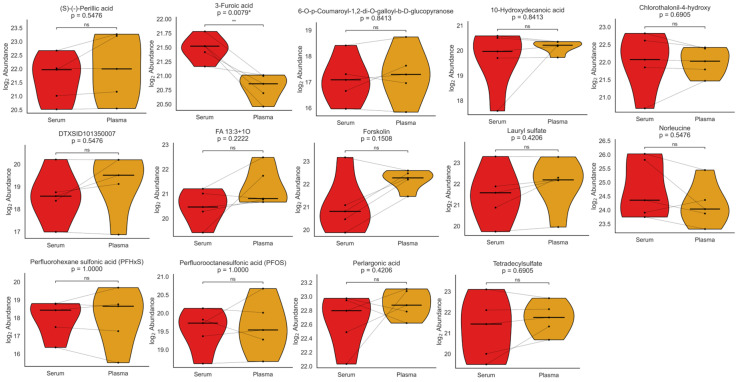
Violin plots of log2-transformed abundance for level 1 and level 2 confirmed exogenous chemicals showing either significant (*p* < 0.05) or no significant difference between serum and plasma, as determined by the Mann–Whitney U test (Method 2, RPLC column, ESI^−^ mode). PFHxS, PFOS, and lauryl sulfate are level 1 confirmed chemicals. Asterisks indicate statistical significance based on the Mann–Whitney U test (** *p* < 0.01) and ns indicates not significant.

For exogenous chemicals detected in ESI^−^ mode (Method 2, RPLC column), a largely consistent pattern was observed, with the majority of the 14 detected compounds (levels 1–2) showing no significant difference in abundance between serum and plasma (Figure 8). Compounds including (S)-(-)-perillic acid, 6-O-p-coumaroyl-1,2-di-O-galloyl-b-D-glucopyranose, 10-hydroxydecanoic acid, chlorothalonil-4-hydroxy, DTXSID101350007, FA 13:3+1O, forskolin, lauryl sulfate, norleucine, perfluorohexane sulfonic acid (PFHxS), perfluorooctanesulfonic acid (PFOS), periargonic acid, and tetradecylsulfate all showed no significant matrix-dependent difference (*p* > 0.05). The only exception was 3-furoic acid, which was significantly higher in serum compared to plasma (*p* < 0.05), representing the sole exogenous chemical in ESI^−^ mode with a statistically significant matrix-dependent difference.

## 4. Discussion

The systematic comparison of serum and plasma in this study revealed consistent yet matrix-dependent differences in metabolite profiles, with implications for study design in NTA metabolomics/exposomics. The clear PCA separation between serum and plasma observed across all three methods, combined with the strong linear correlation, is consistent with prior investigations that while metabolite profiles from plasma and serum were clearly distinct, a high overall correlation (mean r = 0.81 ± 0.10) was observed between the two matrices, indicating that concentration differences are largely systematic rather than random. A contributing factor to these matrix-dependent differences is the use of EDTA as an anticoagulant in plasma collection tubes. EDTA is a well-established source of ion suppression in ESI-based mass spectrometry, where it competes with analytes for charge carriers, forms metal–EDTA adducts, and accumulates at the droplet surface during ionization—collectively reducing the detection efficiency of co-eluting metabolites [25].

The substantial proportion of matrix-unique peak features observed across all methods and ionization modes (Figure 1m–r) likely reflects both biological and analytical differences between serum and plasma. Biologically, the coagulation process during serum preparation releases platelet-derived metabolites and lipid mediators such as thromboxanes and lysophospholipids, which are absent in plasma [12]. Analytically, EDTA in plasma collection tubes chelates divalent metal cations (Ca^2+^, Mg^2+^, Zn^2+^), disrupting metal-dependent metabolite complexes and contributing to ion suppression in ESI-MS [12], particularly affecting features that would otherwise be detectable in plasma. The higher proportion of unique features in ESI^+^ relative to ESI^−^ mode is consistent with the broader susceptibility of nitrogen-containing compounds and lipids to matrix-driven ionization variability. These findings suggest that matrix-unique features are not purely analytical artifacts, but partly reflect genuine biochemical differences between matrices.

Serum showed enrichment of glycerophospholipids and sphingolipids, particularly LPCs, relative to plasma. This pattern is consistent with previous reports [6,26,27]. The elevated LPC and phosphatidylethanolamine (LPE) levels in serum are likely driven by phospholipases released from activated platelets during coagulation, as phospholipases are key enzymes in the generation of LPCs and LPEs. This platelet-driven mechanism is further supported by the fact that almost all molecules significantly upregulated in serum compared to plasma were found to be contained in platelet releasates, confirming that platelet activation during blood coagulation directly contributes to the molecular composition of serum [12].

The enrichment of acylcarnitines, including malonylcarnitine, methylmalonylcarnitine/succinylcarnitine, and 3,4,5-trihydroxypentanoylcarnitine, in serum is consistent with the known release of these compounds from platelets during clot formation. Liu et al. [7] reported that acylcarnitines were among the metabolite classes showing significant differences between serum and plasma, with the majority displaying higher levels in serum. Similarly, Yu et al. [6] reported that arginine had the highest concentration difference between the two matrices, displaying a nearly 50% higher concentration in serum, and attributed this to the release of arginine from platelets during the coagulation process. The differential abundance of oxidized and hydroxylated fatty acids, i.e., hydroxyoctadecadienoic acids (HODEs) and hydroperoxy fatty acids, detected by the RPLC column (method 1) with higher levels in plasma warrants consideration in oxylipin-focused studies. For example, previous study found that Thromboxane B_2_ (TXB_2_) and 12-hydroxyeicosatetraenoic acid (12-HETE), which were among the most abundant eicosanoids released by platelets upon coagulation, were significantly downregulated in serum following acetylsalicylic acid administration, illustrating how platelet-derived oxylipin release during coagulation can confound serum-based lipid mediator measurements [12].

Some polar metabolites, e.g., gamma-glutamylacetamide, N-(2-amino-2-carboxyethyl)-glutamine, and glycerophosphocholine, were enriched in plasma, indicating that certain amino acid derivatives and choline-containing compounds are affected by the coagulation process during serum preparation. Conversely, EDTA in plasma collection tubes chelates divalent metal cations (Ca^2+^, Mg^2+^, Zn^2+^), which can inhibit metalloenzymes and disrupt metal-dependent metabolic pathways, potentially suppressing the detectable concentrations of metal-bound metabolites and amino acid complexes in plasma relative to serum.

One interesting finding is that the higher abundance of certain amino acid derivatives, specifically N-(2-amino-2-carboxyethyl)-glutamine and 2-(bis(2-chloromorpholino)ethyl)amino)acetic acid, in plasma compared to serum in the present study contrasts with the majority of prior reports, which consistently found higher amino acid and amino acid derivative concentrations in serum compared to plasma using targeted LC-MS approaches [6,28,29,30]. This discrepancy may be explained by several factors. First, the compounds identified here are structurally complex amino acid derivatives, rather than proteinogenic amino acids, and may behave differently from the free amino acids typically measured in targeted studies, potentially being subject to matrix-specific protein binding or in vitro degradation during the coagulation process that reduces their apparent abundance in serum. Second, the present study employed an NTA workflow without analyte-specific clean-up, which may yield different matrix suppression profiles compared to targeted methods, potentially amplifying or masking certain compound classes differently across matrices. Third, the small pilot sample size may have introduced variability that influenced the direction of observed differences for low-abundance compounds.

In contrast to the metabolome, the exogenous chemical profiles showed remarkable consistency between serum and plasma across both ESI^+^ and ESI^−^ modes, with the large majority of detected compounds showing no statistically significant matrix-dependent differences (*p* > 0.05). This included structurally diverse compounds such as perfluoroalkyl substances (PFHxS and PFOS, level 1), plant-derived compounds, and pharmaceutical compounds such as acetaminophen (level 1) and buprenorphine (level 2). To our knowledge, no published study has directly compared serum and plasma for broad NTA exposomics or exogenous chemical profiling using LC-MS/MS methods equivalent to those employed here, making direct comparisons with the existing literature difficult. The consistency of PFAS measurements across matrices observed in this study is particularly relevant given their widespread use as biomarkers of environmental exposure, suggesting that either serum or plasma can be used interchangeably for PFAS surveillance studies. Only a small number of exogenous chemicals (level 2) showed significant matrix differences. Acetophenazine and DTXSID90964182 ((S)-4-Hydroxy-2-(2-hydroxypropan-2-yl)-7-methyl-2,3-dihydro-5H-furo [3,2-g]chromen-5-one) were higher in plasma in ESI^+^ mode, while propylparaben was higher in serum in ESI^+^ mode, and 3-furoic acid was higher in serum in ESI^−^ mode. The elevated abundances of acetophenazine and DTXSID90964182 in plasma potentially reflect their relatively higher protein binding, where anticoagulants preserve native protein conformations and prevent clot formation, maintaining the total analyte fraction in solution. In serum, highly protein-bound and lipophilic compounds may be partially sequestered within the fibrin–platelet clot and lost upon centrifugation, leading to underestimation. In contrast, propylparaben’s higher abundance in serum may be attributed to its ester functionality, which renders it susceptible to hydrolysis by plasma esterases that remain active in anticoagulant-preserved plasma but are partially consumed or denatured during the serum clotting process. For 3-furoic acid, measured in ESI^−^ mode, the serum > plasma difference might partly be attributable to anticoagulant-mediated ion suppression rather than true concentration differences, as polyanionic anticoagulants such as EDTA and heparin generate competing anions during electrospray ionization that suppress the [M − H]^−^ signal of small acidic analytes in plasma. Together, these observations highlight that plasma–serum discrepancies are compound-specific and governed by a combination of protein binding, enzymatic activity, and matrix-dependent ionization effects. Given that only 4 out of 23 annotated exogenous chemicals showed significant matrix differences, these preliminary findings tentatively suggest broad comparability between serum and plasma for exposome profiling, though confirmation in larger studies is needed.

## 5. Conclusions and Limitations

This pilot study provides an empirical basis for matrix selection in integrated metabolomics and exposomics workflows using NTA. While serum and plasma yielded broadly comparable chemical profiles, as reflected by strong linear correlations across all three column methods, systematic matrix-dependent differences were evident, particularly in lipid and acylcarnitine sub-categories driven by platelet activation during coagulation.

From a biological fidelity perspective, plasma better preserves the in vivo circulating metabolite composition by minimizing ex vivo biochemical transformations associated with coagulation. The coagulation-induced enrichment of glycerophospholipids, sphingolipids, and amino acid derivatives observed in serum represents a systematic biochemical transformation rather than a true reflection of the circulating metabolome, and is therefore a potential source of bias in mechanistic and untargeted studies where matrix composition directly influences detection and apparent abundance of trace-level compounds. Based on these preliminary findings, plasma might represent a more appropriate single matrix for discovery-oriented metabolomics and exposomics studies within NTA workflows, though validation in larger cohorts is needed before a generalized recommendation can be made.

For exogenous chemical profiling, the two matrices were largely comparable, with only four of 36 annotated compounds showing significant matrix-dependent differences (*p* < 0.05). While these findings provide preliminary evidence of broad comparability between serum and plasma for exposomics screening, conclusions regarding full interchangeability should be interpreted cautiously, as the relatively small number of detected exogenous compounds may partly reflect limited detection sensitivity rather than true equivalence across the broader chemical space.

Several limitations of this study warrant explicit acknowledgment. It should be noted that these findings are derived from a small pilot cohort and are not intended to inform clinical or diagnostic matrix selection, where additional criteria such as reproducibility, pre-analytical robustness, and classification performance are paramount. The question of whether serum or plasma is optimal for clinical deployment remains context-specific and beyond the scope of this study. Furthermore, the sample size of five pre-menopausal women limits generalizability, particularly given known inter-individual variability in coagulation dynamics, platelet activity, and hormonal influences on the metabolome. Future studies with larger and more diverse sample sizes are needed to validate these findings across populations. Additionally, the development of robust cross-matrix normalization strategies will be essential for harmonizing data across multi-cohort and biobank-based investigations where matrix type differs for historical or logistical reasons.

## Data Availability

The original contributions presented in this study are included in the article/Supplementary Materials. Further inquiries can be directed to the corresponding author.

## Supplementary Materials

The following supporting information can be downloaded at https://www.mdpi.com/article/10.3390/toxics14060494/s1. Spreadsheet S1: LC gradient elution programs for 3 different methods; Spreadsheet S2: Abundance, mass, RT of ISTDs and QCs; Spreadsheet S3: Chemical features annotated for method 1-HILIC column, ESI^+^; Spreadsheet S4: Chemical features annotated for method 1-HILIC column, ESI^−^; Spreadsheet S5: Chemical features annotated for method 1-RPLC column, ESI^+^; Spreadsheet S6: Chemical features annotated for method 1-RPLC column, ESI^−^; Spreadsheet S7: Chemical features annotated for method 2-RPLC column, ESI^+^; Spreadsheet S8: Chemical features annotated for method 2-RPLC column, ESI^−^.
